# Options in extracorporeal support of multiple organ failure

**DOI:** 10.1007/s00063-020-00658-3

**Published:** 2020-02-24

**Authors:** W. Huber, A. P. Ruiz de Garibay

**Affiliations:** 1 Klinik und Poliklinik für Innere Medizin II, Klinikum rechts der Isar, Technische Universität München, Ismaninger Str. 22, 81675 München, Germany; 2 ADVITOS GmbH, München, Germany

**Keywords:** Extracorporeal organ support, Renal replacement therapy, Albumin dialysis, Plasma separation, Extracorporeal CO_2_ removal, Extrakorporaler Organersatz, Nierenersatztherapie, Albumindialyse, Plasmaseparation, Extrakorporale CO_2_-Entfernung

## Abstract

Multiorgan failure is among the most frequent reasons of death in critically ill patients. Based on extensive and long-term use of renal replacement therapy, extracorporeal organ support became available for other organ failures. Initially, most of these techniques (e.g. extracorporeal membrane oxygenation, extracorporeal CO_2_ removal [ECCO2R] and extracorporeal liver support) were used as stand-alone single organ support systems. Considering multiple interactions between native organs (“crosstalk”), combined or integrated extracorporeal organ support (ECOS) devices are intriguing. The concept of multiple organ support therapy (MOST) providing simultaneous and combined support for different failing organs was described more than 15 years ago by Ronco and Bellomo. This concept also implicates overcoming the “compartmentalized” approach provided by different single organ specialized professionals by a multidisciplinary and multiprofessional strategy. The idea of MOST is supported by the failure of several recent studies on* single* organ support including liver and lung support. Improvement of outcome by ECOS necessarily depends on optimized patient selection, integrated organ support and limitation of its side effects. This implicates challenges for engineers, industry and healthcare professionals. From a technical viewpoint, modular combination of pre-existing technologies such as renal replacement, albumin-dialysis, ECCO2R and potentially cytokine elimination can be considered as a first step. While this allows for stepwise and individual combination of standard organ support facilities, it carries the disadvantage of large extracorporeal blood volume and surfaces as well as additive costs. The more intriguing next step is an integrated platform providing the capacity of multiple organ support within one device. (This article is freely available.)

## Abstract (de)

Das Multiorganversagen ist eine der häufigsten Todesursachen auf der Intensivstation. Die breite Anwendung der Nierenersatztherapie bei akutem und chronischem Nierenversagen ebnete den Weg für andere extrakorporale Organersatzverfahren. Diese wurden zunächst überwiegend als Einzelorganersatztherapien eingesetzt (extrakorporale Membranoxygenierung, extrakorporale CO_2_-Entfernung [ECCO2R] sowie extrakorporaler Leberersatz). Im Hinblick auf multiple Interaktionen zwischen den Organsystemen („crosstalk“) sind kombinierte bzw. integrierte Organersatzverfahren von großem Interesse. Das Konzept der „multiple organ support therapy“ (MOST) mit kombiniertem Organersatz wurde vor über 15 Jahren von Ronco und Bellomo erstbeschrieben. Dieses Konzept ersetzt den Ansatz der „Kompartimentalisierung“ mit Ersatz einzelner Organversagen im Rahmen der jeweiligen speziellen Verfahren durch eine multidisziplinäre, multiprofessionelle Vorgehensweise. Die Strategie der MOST gewann nach dem Scheitern mehrerer jüngster Studien zum Einzelorganersatz (v. a. Leber- bzw. Lungenersatz) zunehmend an Bedeutung. Der zukünftige Erfolg dieses Konzepts des integrierten Organersatzes hängt dabei auch von einer optimierten Patientenauswahl und einer Limitierung von Nebenwirkungen des Verfahrens ab. Dies bringt zwangsläufig Herausforderungen für Ingenieure, Industrie und Heilberufe mit sich. Technisch ist eine bloße Kombination von vorbestehenden Verfahren wie Nieren- oder Leberersatz, CO_2_-Entfernung und ggf. Zytokinelimination nur ein erster Schritt. Auch wenn dies eine schrittweise und individualisierte Kombination von vorhandenen Organunterstützungstherapien bedeutet, ergibt sich daraus der Nachteil eines hohen extrakorporalen Blutvolumens, großer künstlicher Oberflächen und additiver Kosten für die einzelnen Verfahren. Der notwendige nächste Schritt sind integrierte Verfahren, die einen Multiorganersatz in einem Gerät ermöglichen.

## Introduction

Synchronous or sequential failure of different organs has been termed multiorgan dysfunction syndrome (MODS) or multiorgan failure (MOF). It was first described 50 years ago as a syndrome with “respiratory failure, hypotension, sepsis and jaundice” [1]. MOF is the most frequent cause of mortality in critically ill patients [2]. An increasing number of extracorporeal organ support modalities is intriguing to provide extracorporeal organ support (ECOS) [2–6]. This review reports on recent advances in diagnosis and therapy of MOF.

## History of extracorporeal organ support

In the last two decades, experimental research as well as clinical data (e.g. the SOFA database) emphasized that organ failure is rarely a “stand-alone” organ failure [7]. By contrast, combined and interacting organ failures are frequent. While humoral and cellular interaction—termed “organ crosstalk”—has been characterized more recently [3], syndromic combined organ failure has been described for a long time. For example, *hepatorenal syndrome* is associated with a dramatic decrease of survival compared to single organ failure of a *compensated cirrhosis*.

Even if the term extracorporeal organ support has been recently generalized [5], this concept was introduced about 100 years ago, when the first devices for renal replacement therapy (RRT) were investigated. Based on the theories from Graham, and the experiences from Haas and Abel, Rowntree and Turner, RRT became widely available starting in the 1950s and part of clinical routine thanks to the designs from Kollf [8]. Continuous technological improvements permitted the application of intermittent modalities for chronic patients by Scribner in 1960, the treatment of fluid overload by ultrafiltration by Silverstein in 1974, employing what is now known as slow continuous ultrafiltration (SCUF), the first continuous renal replacement therapy (CRRT) by Kramer in 1977 and newer techniques as the slow extended daily dialysis (SLEDD) introduced by Depner and Golper in 1998 [9].

In parallel, extracorporeal support for other organs was developed. Gibbon was the first to use artificial oxygenation and perfusion support for the first successful open-heart surgery in 1953 [10]. Ten years later, Kolobow described the construction and evaluation of an alveolar membrane artificial heart lung [11]. This was “the embryo” of the extracorporeal membrane oxygenation (ECMO), which was first successfully used in treatment by Hill in 1972.

Based on this previous experience, liver-support therapies using albumin dialysis as principle, and CO_2_ removal devices employing membrane oxygenators are now available. Moreover, other add-on devices (e.g. CytoSorb) for the removal of disease mediators during sepsis have also gained attention.

This shows a large battery of therapies available. However, as suggested by other authors [4–6], it is expected that future developments converge into a single device capable of achieving multiorgan support to cover the lung, the heart, the kidney and the liver [5]. In line with this, a landmark animal study characterized already more than 30 years ago the potential hemodynamic impairment as well as the amount of blood flow required for renal replacement, decarboxylation and oxygenation (Table 1; [12]).

**Table 1:** Comparative technical difficulty of hemodialysis, extracorporeal removal of carbon dioxide and extracorporeal oxygenation. (Adapted from Gattinoni et al. [12])

	Renal replacement	CO_2_ Removal	Extracorporeal oxygenation
*Extracorporeal blood flow (mL/min)*	200–300	500–1000	2000–4000
*Blood pumping*	Optional	Optional	Required
*Hemodynamic changes*	Small	Small	Major
*Vascular access*	Small	Intermediate	Large
*Requirement for anticoagulation*	Small	Small	Large

**Fig. 1:**
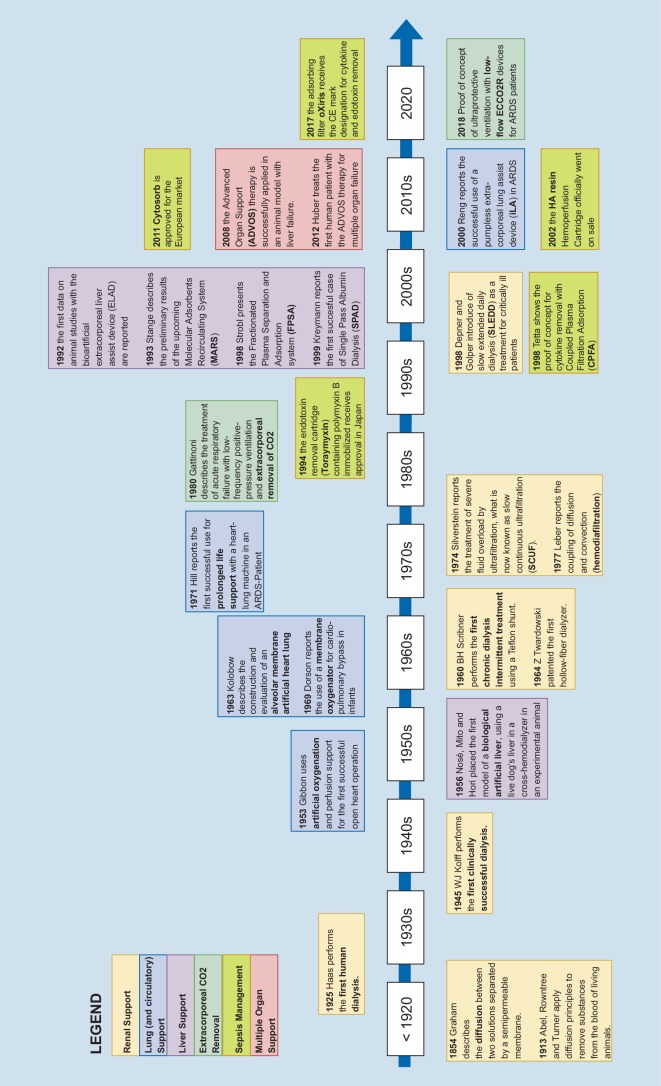
Development of extracorporeal organ support (ECOS). *ARDS* Acute Respiratory Distress Syndrome, *CE* Conformité Européenne

Driven by the “proof of principle” of long-term organ support by chronic hemodialysis, numerous devices for extracorporeal single organ support have been introduced (Fig. 1).

Despite specific features these devices share some common principles and risks (Table 2).

**Table 2:** General principles and risks of extracorporeal organ support (ECOS)

	Risk	Complication
*Extracorporeal circuit*	Volume loss	Hypovolemia; anemia
	Blood flow	Hemodynamic impairment
	Biocompatibility	Allergy, inflammation, immune modulation
	Anticoagulation	Bleeding
	Thermal loss	Hypothermia
*Vascular access*	Vascular damage	Bleeding
	Thrombosis	Venous and arterial embolism
	Blood stream infection	Sepsis

## Characteristics of specific organ support

### Renal replacement

Up to 7% of hospitalized patients develop acute kidney injury [13] during their hospital stay. Among critically ill patients in the intensive care unit (ICU), this rate reaches even 25% [14]. What is more, a mortality rate >50% has been reported for patients with AKI and multiorgan failure [15]. In the absence of any effective pharmacologic therapies, severe AKI can only effectively be managed by RRT.

RRT can be applied with continuous or intermittent modalities. On the one hand, continuous renal replacement therapy (CRRT) refers to any device or technique aiming to replace kidney function for blood purification during an extended period of time. Intermittent therapies are conducted during up to 5 h. A successful CRRT results in a better hemodynamic stability, reduced transcellular solute shifts, and better tolerance to fluid removal. On the contrary, the need of continuous anticoagulation, patient monitoring, alarm vigilance, and experienced staff can be seen as its major disadvantages. On the other hand, during intermittent treatments, an adequate vascular access, specially trained nurses, and continuous pure water supply are demanded. Several forms of RRT can be employed [16]:

- *Slow continuous ultrafiltration (SCUF)* is a continuous therapy that might be used to reach a correction of fluid overload in refractory patients by applying a slow removal of plasma water.
- *Continuous veno-venous hemofiltration (CVVH)* provides solute clearance and volume control by convection. Replacement fluids are infused before or after the hemofilter to replace the ultrafiltrate by predilution or postdilution, respectively.
- *Continuous veno-venous hemodialysis (CVVHD)* uses diffusion for detoxification. This is achieved flowing dialysate into the dialysate compartment of the hemodialyzer either co-currently or counter-currently. IHD refers to intermittent hemodialysis.
- *Continuous veno-venous hemodiafiltration (CVVHDF)* is a combination of the two previous techniques. The intermittent variant is known as intermittent hemodiafiltration (IHDF),
- *Continuous veno-venous high-flux hemodialysis (CVVHFD)* or intermittent high-flux dialysis (IHFD) is a modified hemodialysis where high-flux membranes are applied.

## Extracorporeal lung support: oxygenation

Despite several effective approaches including prone positioning and low tidal volume ventilation, acute respiratory distress syndrome [17] still has a mortality of more than 40% and affects about 10% of ICU patients. Extracorporeal lung support was introduced more than 80 years ago with Gibbon’s heart–lung machine [18]. The first case reports on the clinical use of ECMO in ARDS and preterm infants were published in the 1970s. The first two randomized controlled trials (RCTs) provided the proof of principle with improved oxygenation, but no survival benefit. The lack of improved outcome was mainly due to unacceptably high blood losses and the absence of a lung-protective ventilation under ECMO [19, 20]. Heparin-coating of the ECMO surfaces allowed for a reduction of high-dose heparinization and reduced complication rates in the two more recent RCTs: CESAR and EOLIA [21, 22]. Both trials gave hints on a reduction of mortality by ECMO in selected patients with ARDS. Nevertheless, the improvement of the outcome was lower than assumed for the power calculation in both trials. In fact, the EOLIA trial was stopped for futility despite a nonsignificant 11% reduction in mortality. Both studies and several registries provided important subgroup analyses suggesting several approaches to improve the effect size of ECMO. Among those are a better patient selection and an optimized set-up of the extracorporeal device. Patients with ARDS should be allocated early (i.e. within about 4 days of intubation). Subtle subgroup analyses of EOLIA suggest that ECMO was more beneficial in patients with *less impairment of oxygenation* (pO2/FiO2 ≥66 mm Hg), but *more pronounced hypercapnia* (pCO2 ≥55 mm Hg).

Furthermore, outcome of patients with ECMO therapy is strongly predicted by concomitant nonpulmonary organ failure. In EOLIA, ECMO reduced mortality from 39 to 22% in patients with a SOFA score <11 but was completely ineffective in patients with SOFA ≥11.

This emphasizes the need for improved *multi*organ support. Interestingly, 17% of the patients randomized to ECMO in the CESAR trial (but none of the controls) were treated with the MARS liver support device.

## Extracorporeal lung support: CO_2_ removal

Considering the invasiveness and risks of high-flow ECMO, Gattinoni and coworkers introduced the concept of less invasive extracorporeal lung support restricted to CO_2_ removal (ECCO2R) [23].

With a more limited blood flow, ECCO2R technologies are intriguing for combination with other ECOS devices, in particular with RRT. As shown in Table 3, at least five studies reported on the feasibility of low-flow ECCO2R combined with an ultraprotective ventilation aimed at tidal volumes of 4 instead of 6 ml/kg predicted bodyweight (Table 3).

**Table 3:** Studies on ECCO2R and ultraprotective ventilation

Reference	Device	Number	Main inclusion/exclusion criteria	Additional organ failures	Blood flow; catheter	Period of ECCO2R
Terragni et al. [28]	Decap	10; 22 controls	ARDS (AECC) ≤3 days	SAPS II ca. 48	300–350 mL/min 14 Fr	>72 h
Fanelli et al.[29]	A‑Lung	15	Moderate ARDS (P/F 100–200 mm Hg)	SOFA 10 ± 4	300–350 mL/min 15.5 Fr	3 days
Schmidt et al. [30]	PrismaLung	20	Mild to moderate ARDS (P/F 100–300 mm Hg)	SAPS 56 ± 21 SOFA 9 ± 4	420 mL/min 13 Fr	≥1 day (mean 31 h)
Combes et al. [17]	33 A-Lung 34 iLAactivve 28 Cardiohelp	95	Moderate ARDS (P/F 100–200 mm Hg)	SAPS 46 ± 16 SOFA 7 ± 3	300–500 mL/min (A-Lung) 800–1000 (iLAactivve; CardoHelp)	≥1 day
					Jugular twin-catheter 15.5 Fr (A-Lung) 18.20 Fr (iLA; CardioHelp)	
Nentwich et al. [31]	PrismaLung	20	Hypercapnic acidosis with a pH below 7.30 and a PaCO2 of at least 55 mm Hg under a plateau pressure of at least 25 cmH2O	SOFA 14 (8–18)	Target flow 400 mL/h 13.5 Fr	3 days
ADVOPROTECT (ongoing; Huber et al.)	ADVOS multi	20	Moderate or severe ARDS (P/F ≤200 mm Hg)	No restrictions. At least kidney or liver failure	Target flow 200 mL/h 13 Fr	≥1 day

*ARDS* Acute Respiratory Distress Syndrome, *AECC* American European Consensus Conference, *SAPS* Simplified Acute Physiology Score, *SOFA* Sequential Organ Failure Assessment, *ADVOS* Advanced Organ Support

Finally, pumpless extracorporeal lung assist (pECLA) with a blood flow around 1000 ml/min has been shown to effectively remove CO_2_, while improvement of oxygenation is limited due to the “midrange” blood flow [24, 25].

Regarding *multiorgan support,* some of the ECCO2R devices are prepared for combined use with CVVH(D)F. However, most of these studies (Table 3) excluded patients with other organ failures (in particular liver failure). By contrast, the ongoing ADVOPROTECT trial deliberately includes patients with liver and renal failure.

Another technology of interest has been termed “respiratory electrodialysis”. This procedure combines a hemodiafilter with a membrane lung and a electrodialysis cell cell positioned on the hemodiafiltrate. This technology regionally increases the blood chloride concentration to convert bicarbonate to CO_2_, thus enhancing the CO_2_ extraction by the membrane lung [26, 27].

## Extracorporeal liver support

In addition to the kidneys and lungs, the liver is one of three major detoxification organs. While renal failure results in the accumulation of water-soluble toxins and fluid, liver failure reduces the elimination of protein-bound toxins and liver synthesis.

During the 1990s several extracorporeal methods to eliminate protein-bound toxins were introduced. The most common approach to date is termed albumin dialysis. It is based on the addition of 2–6% albumin to the dialysate to facilitate transport of protein-bound toxins from the blood across the semipermeable membrane into the dialysate. Single-pass albumin dialysis (SPAD) is straightforward but results in a complete waste of the albumin- and toxin-containing dialysate. The proof of principle has been shown in a patient with a serum bilirubin concentration of 102 mg/dL due to liver failure induced by Wilson disease [32]. Although the method is effective for bilirubin and copper removal, the albumin waste results in inacceptable financial burden, particularly, in case of repeated treatment. Therefore, several approaches to “regenerate” the toxin-loaded albumin in the dialysate have been introduced.

### MARS.

The molecular adsorbent recirculating system [33] has been shown to efficiently remove bilirubin as well as ammonia and creatinine. The toxin-loaded albumin in the dialysate is regenerated in a secondary circuit with two adsorption columns (charcoal and an anion-exchange resin). Initial clinical trials suggest improvement of encephalopathy, circulation, portal hypertension and major outcomes. Nevertheless, the largest RCT, the RELIEF trial [34], did not show overall improvement of survival of patients with acute on chronic liver failure (ACLF) [34]. However, a recent subgroup analysis demonstrated an improved 28-day transplant-free survival of patients with ACLF grade two or three [35]. According to the ACLF definition, these were the more severely ill subgroups with at least two or three organ failures. This suggests a potential of MARS for multiorgan support by elimination of water- and protein-bound toxins.

### Fractionized plasma separation and adsorption system (FPSA; Prometheus).

This technology combines separation of toxin-loaded albumin by an albumin-permeable membrane, and removal of the protein-bound toxins through two absorbers (a neutral resin and an anion exchanger) with hemodialysis once the purified plasma returns to the extracorporeal blood circuit. Similar to the RELIEF trial with MARS, also the HELIOS trial with the Prometheus device did not show improvement in survival by extracorporeal FPSA therapy. However,—again—there was a significant survival benefit for the more severely ill patients of the subgroup with a MELD score >30 [36].

### High-volume plasma exchange (HVP).

Plasma separation and replacement with fresh-frozen plasma (FFP) is an established extracorporeal procedure for removing protein-bound toxins. Furthermore, it allows for efficient support of plasmatic coagulation. Several smaller case series gave hints that HVP might improve the outcome in patients with acute liver failure (ALF). A RCT comprising 182 patients with ALF demonstrated significantly improved survival and a significant reduction in the SOFA score and SIRS criteria by HVP [37]. Interestingly, the survival benefit of HVP was greater in those patients who did not undergo emergency liver-transplantation.

### Bioartificial liver (BAL) support.

Extracorporeal bioartificial cellular therapies using extracorporeal liver cell bioreactors for blood purification have been investigated for decades. However, results in patients are still controversial. A recent meta-analysis on 18 clinical trials and 12 preclinical studies, suggested survival improvements are only shown in large animals, but not in humans with ALF [38]. In order to see progress in this area, alternative high-quality liver cells might be necessary, together with well-designed trials, analyzing the effects on subgroups such as primary nonfunction or fulminant hepatic failure. A phase 2 study did not show improved outcome of patients with end-stage liver disease, but demonstrated a trend to better outcome in a subgroup of patients with alcoholic steatohepatitis [39]. A RCT with 203 patients did not demonstrate an improved overall survival in patients treated with the extracorporeal liver assist device (ELAD) compared to standard therapy. Subgroup analyses suggest a potential benefit in younger patients (<47 years) with a MELD score <28 [39].

### Hemadsorption.

A few case reports and small case-series suggest that bilirubin is eliminated by the hemadsorption device CytoSorb [40]. Based on the methodology, so far no conclusions about an improved outcome can be drawn so far.

### Advanced organ support

The advanced organ support (ADVOS) multihemodialysis device is based on the principle of albumin dialysis. The proof of principle has been shown in preclinical studies and case series [41–43]. Beyond the normal renal replacement function, it can eliminate protein-bound substances and CO_2_ [44]. These properties are due to an “intelligent” dialysate: Toxins diffused from blood into the dialysate are eliminated after the application of physicochemical changes (e.g., pH) to the recirculating dialysate in a secondary circuit. This is due to conformational change occurring in albumin above a concrete pH level, which helps both to toxin removal and albumin recycling [13]. In addition, since the dialysate is formed via the on-line mixing of an acidic and an alkaline concentrate, the previously mentioned pH changes can be customized to adapt the dialysate pH. Overall, ADVOS intends to provide a multiple organ (i.e. kidney, liver, lungs) support by means of water-soluble, protein-bound toxins elimination, direct H^+^ removal (i.e. acid–base balance) and CO_2_ elimination.

Serum albumin, is the main protein of human blood plasma. It binds, among others, fatty acids, hormones or bilirubin. An increase of the latter 5 times above the upper limit increases the risk to develop cholemic nephropathy [45–47]. Furthermore, new onset of acute kidney injury is associated with concomitant onset of jaundice [48]. The reduction of bilirubin levels (ideally by normalization of the hepatic function, alternatively by extracorporeal detoxification) by the ADVOS multi device has been shown in several studies. On top of this, as already documented [43], ADVOS multi can remove creatinine, urea or ammonia, among others.

Nevertheless, probably, the most differentiating factor of the ADVOS therapy in comparison to other apparently similar medical devices is the possibility to adjust the pH of the dialysate (by the relation between the acidic and basic concentrates that form the dialysate) and adapt it to the needs of the patient during treatment. Going back to chemistry basics, when the pH of a solution is higher than 7.00, the concentration of OH^−^ is likewise higher than that of H^+^. The higher the pH of the dialysate, the higher the gradient of H^+^ that can be formed between blood and dialysate. Consequently, H^+^ in excess will diffuse from blood into the dialysate, providing an acidosis correction. Moreover, by removing H^+^, HCO_3_^−^ will be produced in blood (Eq. 1), mimicking the mechanism used by the kidney as a metabolic response to respiratory acidosis.

#### Equation 1.

Equilibrium reaction between CO_2_, H^+^ and HCO_3_^−^

1 $$\mathrm{CO}_{2}+\mathrm{H}_{2}\mathrm{O}\leftrightarrow \mathrm{H}_{2}\mathrm{CO}_{3}\leftrightarrow \mathrm{HCO}_{3}^{-}+\mathrm{H}^{+}$$

The generated HCO_3_^−^ provides an improvement during metabolic acidosis, but should be removed, if excessive, during respiratory acidosis. The capacity of the ADVOS system to remove CO_2_ depends on blood flow, dialysate pH and the bicarbonate concentration. As demonstrated in a series of experiments using an ex vivo model for acidosis, the higher the dialysate pH, the blood flow or the accumulated HCO_3_^−^, the better CO_2_ removal rates are achieved [44]. In the clinical setting ADVOS is normally used with a maximum blood flow rate of 200 ml/min (to allow regional citrate anticoagulation), a maximum dialysate pH of 9 and basic concentrates containing 20 mmol/l HCO_3_^−^. This allows a removal of up to 50 ml/min CO_2_ with normal blood bicarbonate concentration (22–28 mmol/l). Since the HCO_3_^−^ removal is the limiting factor in the ADVOS multi circuit, during a severe metabolic acidosis even more CO_2_ could be removed without an increase of blood bicarbonate over 30 mmol/l. Under experimental conditions, up to 146 ml/min of CO_2_ could be removed. However, this required blood flow rates of 400 ml/min and a dialysate pH >9.00 with a basic concentrate without bicarbonate [44].

In contrast to ECMO, where due to high blood flows (3–6 L/min) blood pH is normalized within minutes, it takes up to 2–4 h for ADVOS multi running at 100–200 ml/min blood flows until an acidotic blood is normalized in patients. The use of elevated dialysate pH is not exempt of risks, and therefore, to avoid overcompensation, blood pH must be continuously monitored during ADVOS treatments. It is recommended that blood pH values of the samples taken at the outlet of the dialyzer (blood post-dialyzer) remain below 8.00. Above this value pH is no longer measurable in common blood gas analyzers. In case that a post-dialyzer blood pH is >8.00, dialysate pH should be reduced by 0.5 in the treatment’s settings (e.g., from 9.00 to 8.50).

Table 4 summarizes the main features of clinically available devices for extracorporeal liver support.

**Table 4:** Summary of features of clinically available devices for extracorporeal liver support

	Liver support	Renal support	ECCO2R	Acid–base modulation	Improved coagulation	Resources required	Availability	Financial burden
*SPAD*	+	+	−	−	−	+++	++	+++
*MARS*	+	+	−	−	−	+++	+	+++
*PROMETHEUS*	+	+	−	−	−	+++	+	+++
*ADVOS*	+	+	+	+	−	++	+	+++
*ELAD*	+	+	−	−	−	++++	−	++++
*Plasma separation*	+	−	−	−	+	++	++	++
*CytoSorb*	+	−	−	−	−	++	+++	++

*SPAD* Single Pass Albumin Dialysis, *MARS* Molecular Adsorbent Recirculating System, *ADVOS* Advanced Organ Support, *ELAD* Extracorporeal Liver Assist Device

## Detoxification in sepsis

Major parts of the pathophysiology of sepsis are related to microbial toxins and to the inflammatory response induced by proinflammatory cytokines. Therefore, extracorporeal elimination of toxins and cytokines is an intriguing concept to treat patients with sepsis.

In the first case, hemoperfusion using fiber columns containing polymyxin B (an antibiotic with high affinity to endotoxins) has been used in a number of studies. However, recent results and meta-analyses did not demonstrate improved outcome by this or similar approaches [33, 49–51].

In the second case, CytoSorb provides hemoadsorption of cytokines and other midmolecular weight toxins by multiple porous polymeric beads. Two larger studies in septic patients resulted in conflicting data: A RCT including 100 mechanically ventilated patients with severe sepsis or septic shock did no show a reduction in systemic IL‑6 levels or in multiple organ dysfunction score, ventilation time and time course of oxygenation in the intervention group [52]. A retrospective analysis of 116 patients with septic shock demonstrated a significantly higher reduction in predicted mortality in patients with CytoSorb therapy and CRRT compared to patients with CRRT alone [53].

Similarly, the HA 330 and HA 380 cartridges (Jafron, Zhuhai, China) contain neutro-macroporous resin adsorbing beads with a pore size of 500 D–60 kD. At least two RCTs with 44 and 46 patients demonstrated significantly improved outcome (including ICU mortality) in patients treated with HA 330 hemoperfusion [54, 55].

## Modular or integrated multiorgan support?

While there is increasing evidence for combined MOST, there is an ongoing debate about its realization. From a pragmatic viewpoint individual combination of the available devices is a first reasonable step. In particular, liver support systems such as MARS and Prometheus, and some devices for ECCO2R are usually combined with sequential RRT devices. Furthermore, the high blood flow during ECMO allows for RRT in parallel without additional vascular access [56].

Nevertheless, modular combination results in additional extracorporeal volume and potential hemodynamic impairment. Also regarding fluid balance targets, thorough monitoring of these side effects is mandatory. This starts with the observation of potential hemodynamic impairment during connection and ends with documentation of circulatory changes during disconnection. Several studies showed that transpulmonary thermodilution (TPTD) is feasible during RRT and ADVOS treatments [56]. Despite concerns on a loss of indicator into the extracorporeal circuit, a recent study demonstrated that measurement of Cardiac Index with TPTD is reliable even during ECMO [57], whereas global end-diastolic volume index (GEDVI) and extravascular lung water index (EVLWI) might be confounded.

Regarding the disadvantages and technical burdens of using combinations of pre-existing technologies (Table 5), development of procedures facilitating MOST by one single device is an intriguing next step. Although there is still a lack of data on improved outcome, ADVOS can be considered as the first integrated MOST device.

**Table 5:** Comparison of combined single organ support and multiorgan support devices

Combination of single organ support devices		Multiorgan support devices	
Advantage	Disadvantage	Advantage	Disadvantage
Step-wise combination	Large extracorporeal volume	Limitation of extracorporeal volume	Not yet generally available
Use of familiar technique	Personal resources for assembling several devices	Limitation of personal resources	Little clinical data available
	Cumulative costs of several devices	Additional features: modulation of acid–base balance	
	Lack of “match-up”		

## Practical conclusion

During the last few decades, extracorporeal organ support has become available for nearly every organ failure. All types of ECOS share the challenges of vascular access, sequestration of blood into the device, induction of extracorporeal blood flow, anticoagulation with potential bleeding or clotting complications, a certain circulatory impairment, and finally, the attempt of extracorporeal blood purification.

Based on organ-specific compensatory mechanisms and blood flow within the genuine organ(s), extracorporeal blood flow ranges from below 100 ml/min up to more than 5 l/min in ECMO. Due to the high incidence of MOF in critically ill patients, the concept of multiorgan support is intriguing. Depending on the individual organ failures, in some patients, multiorgan support can be provided by sequential and/or intermittent therapy with single-organ support systems. Another option is combined organ support (normally two organ support) using serially connected devices driven by one blood pump. Considering the additive sequestration of blood in several devices, integrated multiorgan support using one multifunctional device might be the most intriguing approach.
